# Evidence for varicose vein treatment: an overview of systematic reviews

**DOI:** 10.1590/1516-3180.2018.0003240418

**Published:** 2018-07-16

**Authors:** Ricardo de Ávila Oliveira, Andréa Castro Porto Mazzucca, Daniela Vianna Pachito, Rachel Riera, José Carlos da Costa Baptista-Silva

**Affiliations:** I MD, MSc. Vascular Surgeon, Adjunct Professor, Universidade Federal de Uberlândia (UFU), Uberlândia (MG), and Postgraduate Student in the Evidence-Based Health Program, Universidade Federal de São Paulo (UNIFESP), São Paulo (SP), Brazil.; II BSc. Pharmacist and Postgraduate Student in the Evidence-Based Health Program, Universidade Federal de São Paulo (UNIFESP), São Paulo (SP), Brazil.; III MD, MSc. Neurologist and Postgraduate Student in the Evidence-Based Health Program, Universidade Federal de São Paulo, (UNIFESP), São Paulo (SP), Brazil.; IV MD, PhD. Rheumatologist, Assistant Professor of the Discipline of Evidence-based Health, Escola Paulista de Medicina, Universidade Federal de São Paulo (UNIFESP), and Assistant Coordinator at Cochrane Brazil, São Paulo (SP), Brazil.; V MD, PhD. Full Professor of the Discipline of Vascular Surgery, Universidade Federal de São Paulo (UNIFESP), São Paulo (SP), Brazil.

**Keywords:** Varicose veins, Sclerotherapy, Laser therapy, Surgical procedures, operative, Review [publication type]

## Abstract

**BACKGROUND:**

Varicose veins affect nearly 30% of the world’s population. This condition is a social problem and needs interventions to improve quality of life and reduce risks. Recently, new and less invasive methods for varicose vein treatment have emerged. There is a need to define the best treatment options and to reduce the risks and costs. Since there are cosmetic implications, treatments for which effectiveness remains unproven present risks to consumers and higher costs for stakeholders. These risks and costs justify conducting an overview of systematic reviews to summarize the evidence.

**DESIGN AND SETTING:**

Overview of systematic reviews within the Discipline of Evidence-Based Health, at Universidade Federal de São Paulo (UNIFESP).

**METHODS:**

Systematic reviews on clinical or surgical treatments for varicose veins were included, with no restrictions on language or publication date.

**RESULTS:**

51 reviews fulfilled the inclusion criteria. Outcomes and comparators were described, and a narrative review was conducted. Overall, there was no evidence that compression stockings should be recommended for patients as the initial treatment or after surgical interventions. There was low to moderate evidence that minimally invasive therapies (endovenous laser therapy, radiofrequency ablation or foam sclerotherapy) are as safe and effective as conventional surgery (ligation and stripping). Among these systematic reviews, only 18 were judged to present high quality.

**CONCLUSIONS:**

There was evidence of low to moderate quality that minimally invasive treatments, including foam sclerotherapy, laser and radiofrequency therapy are comparable to conventional surgery, regarding effectiveness and safety for treatment of varicose veins.

## INTRODUCTION

Varicose veins are enlarged and tortuous veins.^1^ They are part of the chronic venous insufficiency syndrome^2^ and are associated with complications such as edema, skin pigmentation, lower-limb ulcers, thrombophlebitis and bleeding.^3^ This clinical variability has led to use of a classification system for chronic venous disorders (CEAP), as follows: C0 (no varicose veins); C1 (telangiectasias and reticular varicose veins up to 4 mm in diameter); C2 (trunk varicose veins); C3 (edema relating to varicose veins); C4 (skin pigmentation); C5 (healed venous ulcer); and C6 (active venous ulcer).^2^ Eklöf revised the CEAP classification, including modification of the threshold for reticular varicose veins from 4 mm in diameter to a maximum of 3 mm.^4^ However, there is no absolute consensus regarding the classification of varicose veins, which imposes limitations on comparisons of results between different studies.^5^

The prevalence of varicose veins reaches up to one-third of the Western population.^3^ Prevalence rates vary due to different definitions in epidemiological studies, ranging from less than 1% to 73% among women, and from 2% to 56% among men.^6^ In Brazil, the prevalence rate reaches around 50%, after excluding CEAP C1.^7^^,^^8^ Lower-limb ulcers affect 1-2% of the world’s population, and this has clinical and economic impacts.^8^^,^^9^

Treatment of varicose veins can be justified by its positive impact on quality of life.^3^ The financial burden due to venous ulcers in the United States has been estimated to be 14.9 billion American dollars a year.^10^ Moreover, because esthetic concerns impose a need for treatment, such concerns may lead to institution of ineffective and potentially harmful treatments. In Brazil, the cost of treatment increased four-hundredfold between 1995 and 2001.^8^

The high prevalence of this disease, the costs, the potential for complications attributed to its treatment and the need to disseminate science among stakeholders justify conducting a high-quality synthesis of systematic reviews on this topic, with the aim of mapping out the current knowledge and identifying gaps in the literature to guide future sound research.

The primary objective of this study was to summarize evidence derived from systematic reviews focusing on interventions to treat varicose veins. In addition, the following secondary objectives were defined:

1. To describe comparisons applied in studies;
2. To verify outcomes chosen to evaluate treatment;
3. To assess the methodological quality of systematic reviews on the topic;
4. To describe the strength of evidence according to different outcomes.

## METHODS

This study was an overview of systematic reviews, conducted within the Discipline of Evidence-Based Health, at the Federal University of São Paulo (Universidade Federal de São Paulo, UNIFESP).

The inclusion criterion for the systematic reviews was that they needed to focus on clinical or surgical interventions for lower-limb varicose veins, provided that the abstracts contained the terms systematic review and/or meta-analysis and that a full report was available. In cases of updates of the same review, only the most recent version was considered for inclusion. The following types of study were excluded: narrative reviews, conference proceedings, structured abstracts and systematic reviews focusing on the healing of lower limb ulcers without venous interventions.

A search strategy was run in the following databases: MEDLINE, EMBASE, LILACS and CENTRAL (last updated on September 3, 2017), applying the terms “varicose veins” or “varices” or “telangiectasias”. Regarding the LILACS database, 286 references were retrieved using the term “varicose veins” and synonyms, thus dispensing with the need for filters. For all other databases, a filter that had been developed for retrieval of systematic reviews was used. There were no limitations regarding language or publication date. We conducted a hand search of references presented in the studies included in our review.

Two authors independently screened studies (RAO and ACPM), and any disagreements were resolved by a third author (RR), through use of Rayyan software.^11^ Two independent authors conducted data extraction (RAO and ACPM), and disagreements were resolved by reaching a consensus.

The AMSTAR tool (assessment of multiple systematic reviews) was applied to assess the methodological quality of the systematic reviews included.^12^ This tool encompasses 11 items for methodological evaluations, each scoring from 0 to 1. Studies with a total score of 0 to 4 were considered to present low methodological quality; 5 to 8, moderate quality; and 9 to 11, high quality.^13^

## RESULTS

**Figure 1:**
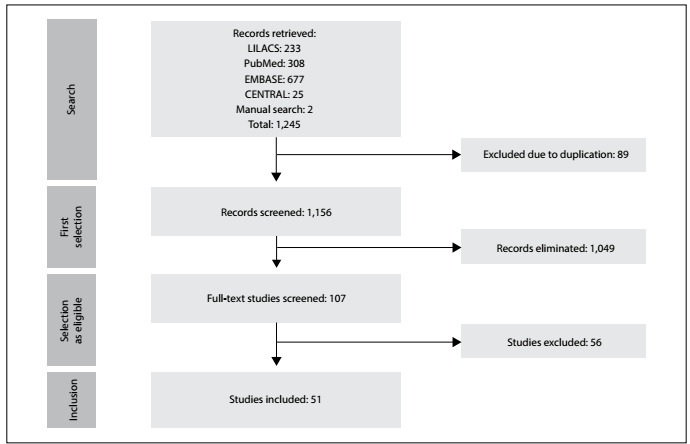
PRISMA flow chart for study selection process.

The search strategy yielded 1,245 studies. 107 studies were considered for inclusion after screening of titles and abstracts, with further retrieval of full texts. Among these, 51 reviews fulfilled the inclusion criteria (Figure 1).

The reviews included were combined into 13 distinct groups of interventions, which were described as follows:

1. Clinical treatment of varicose veins: Amsler and Blättler concluded that compression levels of 10 to 15 mmHg are effective in treating chronic venous insufficiency, despite the weakness of evidence due to heterogeneity across studies.^14^ Two studies suggested that the effectiveness of compression stockings is overestimated, since adherence to treatment under real-world conditions is low, only reaching around 37% of the patients.^15^^,^^16^ Thus, it was claimed that there was no high-quality evidence to support use of compression stockings as the initial type of treatment. Smyth et al. found that rutosides, reflexology and water immersion improved the symptoms in pregnant women with edema relating to varicose veins, although those findings were only based on a moderate level of evidence.^17^ Boada and Nazco concluded that use of venotonics might alleviate the symptoms of fatigued legs. However, the quality of evidence was not assessed.^18^
2. Techniques and complications relating to sclerotherapy: Foam sclerotherapy is effective and safe, although the quality of studies has been considered to be low.^19^ Cerebrovascular events associated with foam sclerotherapy are a rare but still a possible complication that has mostly been reported in the form of case reports.^20^^,^^21^ These side effects seem to be mild, considering that it has been reported that the majority of patients were discharged from hospital without neurological sequelae. One study evaluated sclerosing agents to treat telangiectasias and concluded, based on very low-quality evidence, that one particular agent is not superior to another.^22^
3. Liquid versus foam sclerotherapy: Foam sclerotherapy increases the technical success rates (venous occlusion), in comparison with liquid sclerotherapy.^23^ The quality of the evidence for this finding was not assessed in that report. Despite methodological limitations to evaluations on appropriate methods, dosages, formulations and compression levels, the current evidence supports the use of sclerotherapy in clinical practice.^24^
4. Surgical techniques: The CHIVA technique (ambulatory conservative hemodynamic correction of venous insufficiency) reduces disease recurrence in comparison with ligation and stripping and has been correlated with fewer adverse events.^25^ These findings are based on a few studies with high risk of bias, because of the impossibility of blinding and the small number of incidents reported. Better esthetic results are achieved through use of transillumination, but with a higher number of hematomas and more intense pain in the postoperative period.^26^ The quality of evidence for these findings was not assessed in that report. Studies with high risk of bias have suggested that use of tourniquets reduces bleeding.^27^ Mumme et al. described the valvuloplasty technique and concluded that it was suitable for preserving veins in specific patients who were at high risk of atherosclerotic disease. The quality of the evidence was not assessed.^28^ Pearson et al. took the view that surgery should continue to be used to treat varicose veins in public healthcare systems, although without indicating the most cost-effective technique.^29^ Due to the methodological limitations of the primary studies in that review, no meta-analysis was conducted. Rudström et al. assessed complications relating to the surgical approach and found that despite their infrequency, they were potentially harmful. The most common complication was bleeding after injury to the femoral vein or arterial lesions. The quality of the evidence was not appraised.^30^
5. Surgery versus sclerotherapy: There was no evidence that one treatment was superior to any other. However, it was suggested that sclerotherapy was associated with lower cost of treatment and better results after one year of follow-up.^31^ Surgical outcomes are long-lasting, but it is unknown whether sclerotherapy outcomes also are. The overall quality of the studies included was considered low, mostly due to inadequate randomization. Complications relating to sclerotherapy were infrequent, but the data were deemed to be insufficient for conclusions to be drawn, and the methodological quality of the primary studies was considered low.^32^
6. Surgery versus endolaser therapy (EVLT): All studies concluded that EVLT was as safe as conventional surgery. Van den Bos et al.^33^ and Darwood and Gough^34^ found that rare but potentially harmful complications might be associated with EVLT treatment. The mild complications included ecchymosis, pain, superficial thrombophlebitis, nerve lesion, arteriovenous fistula and matting. The wavelengths applied in EVLT treatment ranged from 810 to 1320 nm, and these were associated with recanalization in 5% of the patients in the first year.^34^ Liu et al.^35^ and Pan et al.^36^ concluded that the results from the two types of treatment were similar over a follow-up period of two years when fibers of 810 nm and 980 nm were used. The quality of the evidence was not appraised. Pan et al.^36^ found that technical failure (saphenous reflux) was more frequent with EVLT, while Xiao et al.^37^ concluded that there were no differences in the results from EVLT and conventional surgery. Risk of bias was assessed in this study, but not the quality of the evidence. Hoggan et al.^38^ and Mundy et al.^39^ came to contradictory conclusions, based on evidence that was of low quality because of ineffective randomization and losses during the follow-up.^38^ Hoggan et al.^38^ concluded that the rates of reflux resolution were comparable, and Mundy et al.^39^ pointed out that EVLT was associated with higher rates of recanalization. Similarly, Lynch et al.^40^ reported that there was a higher risk of recanalization over a twelve-month period, although EVLT was less frequently associated with nerve lesions, infections and skin pigmentation. The findings of that study were based on low-quality evidence. Ruiz-Aragón et al.^41^ also reported that there were fewer complications in the EVLT group, although it was assumed that a risk of bias existed due to exclusion of unpublished studies.
7. Surgery versus radiofrequency ablation (RFA): Radiofrequency ablation was found to be beneficial over the short term, due to lower risk of ecchymosis, hematoma and pain, a more positive impact on quality of life and faster return to work.^42^ On the other hand, radiofrequency ablation increased the risk of recanalization after 12 months.^42^ It was noteworthy that there was no reliable evidence supporting superiority of radiofrequency ablation over conventional surgery.^43^ The rates of complications like deep venous thrombosis reached 1.8%, and recurrence remained to be clarified. Patient satisfaction and preference were found to favor surgery. In Canada, the costs of radiofrequency ablation were considered lower, based on evidence of low to moderate quality.^44^
8. Surgery versus thermal ablation (EVLT or RFA): Conventional surgery and thermal ablation were found to share comparable results over the long term,^45^ with no difference in recurrent rates.^46^ Compared with surgery, thermal ablation was considered safe and effective, with the advantage of being associated with faster recovery over the short and medium terms.^47^ The quality of evidence was not appraised in any of these studies.
9. EVLT versus RFA: The outcomes were considered comparable over the short term^48^ and over a longer term of five years.^49^ He et al.^48^ concluded that the quality of evidence to support these findings was low, while Balint et al.^49^ did not appraise the quality of evidence.
10. Surgery versus EVLT, RFA or foam sclerotherapy: Van den Bos et al., Nesbitt et al., and Leopardi et al.^50^^,^^51^^,^^52^ considered that minimally invasive techniques were as effective and safe as surgery. Thermal ablation was considered superior to surgery.^53^ According to Murad et al.,^54^ surgery and minimally invasive treatments were safe and effective, although minimally invasive procedures resulted in less disability and postoperative pain. Carrol et al.^55^ concluded that alternative therapies were a possible substitute for surgery, and pointed out that foam sclerotherapy was probably more cost-effective. Paravastu et al.^56^ found that the rate of recanalization of the small saphenous vein over the short term was higher in the conventional surgery group than in the EVLT group, and that the rate was uncertain for foam, compared with surgery. Overall, the quality of evidence either was considered low due to the small number of events and use of surrogate outcomes or was not appraised.
11. Compression versus surgery for leg ulcers: One author considered compression to be the first-line treatment for leg ulcers.^57^
12. Surgery for leg ulcers: Samuel et al.^58^ did not identify any clinical trial. Mauck et al.^59^ recommended surgery and considered that surgical treatment might improve healing. This finding was mostly based on observational studies. According to Howard et al.,^60^ surgery was associated with rates of healing similar to those for compression alone, but presented lower levels of recurrence. The quality of evidence was not assessed.
13. Any postoperative intervention: Postoperative compression may reduce the extent of hematomas and incidence of thrombophlebitis in treatments for telangiectasias and reticular veins over a three-week period.^61^ Conversely, Huang et al.^62^ concluded that compressive therapy lasting for more than seven days was not associated with clinical benefits regarding pain, edema, complication rate and absenteeism. In two studies by El-Sheikha et al.,^63^^,^^64^ no meta-analysis could be conducted because of substantial heterogeneity. Overall, the quality of evidence was either considered low or was not appraised.

The methodological quality of the systematic reviews described above was appraised through using the AMSTAR tool.^12^ Out of these 51 reviews, 18 presented high methodological quality, 21 were of moderate quality and 12 were of low quality (Annex 1).

**Annex 1:** Critical appraisal of studies included, through using the Assessment of Multiple Systematic Reviews (AMSTAR) tool.^12^

Review question	First author	AMSTAR											Total Score	Overall quality
		1	2	3	4	5	6	7	8	9	10	11		
Clinical treatment	Amsler^14^	0	U	0	U	1	1	0	1	0	0	1	4	L
	Palfreyman^15^	0	U	1	1	1	1	0	1	0	0	1	6	M
	Shingler^16^	1	1	1	1	1	1	1	1	1	NA	1	10	H
	Smyth^17^	1	1	1	1	1	1	1	1	1	NA	1	10	H
	Boada^18^	0	1	1	0	1	1	1	1	0	0	0	6	M
Techniques and complications relating to sclerotherapy	Rathbun^19^	0	1	1	0	1	0	0	0	0	0	1	4	L
	Sarvananthan^20^	0	1	1	1	1	1	1	1	1	NA	1	9	H
	Willenberg^21^	0	U	0	1	1	1	0	0	0	0	0	3	L
	Schwartz^22^	1	1	1	1	1	1	1	1	1	1	1	11	H
Foam versus liquid sclerotherapy	Hamel-Desnos^23^	0	U	1	1	1	1	0	0	0	0	0	4	L
	Tisi^24^	1	0	1	1	1	1	1	1	1	1	1	10	H
Surgical techniques	Bellmunt-Montoya^25^	1	1	1	1	1	1	1	1	1	NA	1	10	H
	Luebke^26^	0	0	1	U	1	0	1	0	1	0	0	4	L
	Rigby^27^	1	1	1	1	1	1	1	1	1	NA	1	10	H
	Mumme^28^	0	0	0	0	0	1	0	0	0	0	0	1	L
	Pearson^29^	0	U	1	U	0	1	1	1	1	0	0	5	M
	Rudström^30^	0	U	0	0	0	0	0	0	0	0	0	0	L
Surgery versus sclerotherapy	Rigby^31^	1	1	1	1	1	1	1	1	1	0	1	10	H
	Jia^32^	0	1	1	1	1	1	1	1	1	0	1	9	H
Surgery versus endolaser	Van Den Bos^33^	0	U	U	0	0	0	0	0	0	0	1	1	L
	Darwood^34^	0	U	0	0	1	1	0	0	0	0	1	3	L
	Liu^35^	0	1	1	1	1	1	1	1	1	0	0	8	M
	Pan^36^	0	U	1	1	1	1	1	0	1	0	0	6	M
	Xiao^37^	0	1	1	0	1	1	1	0	1	1	0	7	M
	Hoggan^38^	1	1	1	1	0	1	1	1	0	0	1	8	M
	Mundy^39^	0	1	1	1	1	0	1	1	0	0	0	6	M
	Lynch^40^	1	U	1	1	1	1	1	0	1	0	1	8	M
	Ruiz-Aragón^41^	0	1	1	1	1	1	1	1	0	1	1	9	L
Surgery versus radiofrequency	Luebke^42^	0	U	1	1	1	1	1	0	1	1	1	8	M
	Goodyear^43^	0	U	0	U	0	0	0	0	0	0	1	1	L
	Health Quality Ontario^44^	0	U	1	0	1	1	1	0	0	0	1	5	M
Surgery versus thermal ablation (laser and radiofrequency)	Xenos^45^	0	1	1	1	1	1	0	0	1	1	0	7	M
	O’Donnell^46^	0	1	1	1	1	1	0	0	0	0	1	6	M
	Brar^47^	0	0	1	0	1	1	1	0	1	0	0	5	M
Endolaser versus radiofrequency	He^48^	0	1	1	0	1	0	1	1	U	1	1	7	M
	Balint^49^	0	1	0	0	1	1	0	0	1	1	1	6	M
Surgery versus laser or radiofrequency or foam sclerotherapy	van den Bos^50^	0	1	1	0	1	0	1	0	0	0	1	5	M
	Nesbitt^51^	1	1	1	1	1	1	1	1	1	1	1	11	H
	Leopardi^52^	1	1	1	1	0	1	0	0	0	0	1	6	M
	Boersma^53^	0	1	1	0	1	1	1	0	1	0	1	7	M
	Murad^54^	1	1	1	1	1	1	1	1	1	0	1	10	H
	Carrol^55^	1	1	1	1	1	1	1	1	1	0	1	10	H
	Paravastu^56^	1	1	1	1	1	1	1	1	1	1	1	11	H
Compression versus surgery for lower-limb ulcers	de Carvalho^57^	0	U	1	0	1	1	0	0	0	0	1	4	L
Lower-limb ulcers	Samuel^58^	1	1	1	1	1	1	1	1	1	1	1	11	H
	Mauck^59^	0	1	1	1	1	1	1	1	1	1	1	10	H
	Howard^60^	0	U	1	1	1	1	0	0	0	0	1	5	M
Any postoperative intervention	Noppeney^61^	0	U	0	0	1	1	0	0	0	0	0	1	L
	Huang^62^	0	1	1	1	1	1	1	1	1	0	1	9	H
	El-Sheikha^63^	0	1	1	1	1	1	1	1	1	NA	1	9	H
	El-Sheikha^64^	0	1	1	1	1	1	0	0	0	0	1	6	M

H = high methodological quality; M = moderate methodological quality; L = low methodological quality; NA = not applicable; u = unclear. Total score of 0 to 4 was considered to represent low methodological quality; 5 to 8, moderate quality; and 9 to 11, high quality.^12^

### Potential bias in conducting this overview

No study protocol was developed a priori for this analysis. However, we followed the goals and methods that were initially planned.

No additional search was conducted in the gray literature. However, we did conduct a hand search of references presented in the studies included in our review.

There may also be bias in relation to endolaser technology if studies using interventions at different stages of its development are compared.

## DISCUSSION

This overview revealed heterogeneity in relation to many aspects of varicose disease, including terminology and classification. While some authors described varicose veins as enlarged veins of more than 3 mm in diameter,^4^ others defined them as veins larger than 4 mm in diameter^2^ or included telangiectasias and reticular veins within the definition.^5^ There is still a need for standardization of terminology.^65^

Regarding prophylactic issues, Robertson et al.^66^ did not find any good-quality studies that would enable assessment of whether lifestyle modifications might be useful as prophylaxis and for avoiding complications of varicose veins. Governments should prioritize topics like this when considering which studies to fund, since this issue may have practical implications at low cost, both for individuals and for healthcare systems.

Studies on surgical interventions frequently focus on ideal patients (with uncomplicated varicose veins of limited diameter, saphenous veins that are not very tortuous and absence of previous thrombophlebitis). In real life, patients present heterogeneous disease concomitantly in the same limb. Therefore, there is frequently a need to make use of a combination of techniques to achieve the best results,^31^ based on the characteristics and clinical presentation of the varicose veins.^52^ It is crucial to establish criteria for choosing the most suitable technique for different clinical scenarios.^45^

Sclerotherapy is currently considered to be the first-line treatment for telangiectasias. Other therapies have been proposed as alternatives, but evidence to justify their choice is sparse and indirect.^16^^,^^52^^,^^55^ In fact, surrogate outcomes are frequently reported in trials. Thus, conclusions are based solely on technical parameters^38^^,^^67^ for heterogeneous populations^68^ with short follow-ups,^54^ which serves to increase the uncertainties rather than to resolve them.

Ligation and stripping are frequently chosen as the comparator because of their safety, effectiveness, cost issues and time span, and these have been used as a gold standard.^55^ The complications associated with surgery include nerve lesions, hematomas, postoperative pain and pigmentation. However, severe complications are rare.^30^

Minimally invasive treatments have been developed with the aim of reducing the risks and discomfort, as well as for reducing the time taken to return to work and optimizing cost-effectiveness. Their efficacy and effectiveness are comparable to those obtained through conventional surgery, regardless of the parameters chosen for this comparison. Minimally invasive therapies or surgery cannot always be applied to particular patients.^60^

However, foam sclerotherapy seems to be particularly useful in this context since it can be used alone or in combination with other interventions. For instance, it may improve the results after surgery, bearing in mind that no surgical technique is capable of eliminating all varicose veins. The limitations associated with foam sclerotherapy include higher risk of recanalization and pigmentation,^56^ along with the need for multiple sessions in order to obtain satisfactory results. These restrictions are surpassed by the benefits regarding cost-effectiveness.^55^ We therefore considered it odd that we did not find any studies focusing on foam sclerotherapy for leg ulcers. Since fibrotic tissue may prevent the possibility of stripping some varicose veins, which consequently could maintain the pathological condition and hence the ulcers, foam sclerotherapy might potentially be a better treatment for this population.

There is no evidence that compressive stockings might bring benefits for patients with primary varicose veins.^15^^,^^16^ Questions arise regarding the technical attributes of stockings (the type of elastic material and level of compression), the anatomical characteristics of the lower limbs and patients’ mobility while using these stockings.^69^ Furthermore, there is low compliance due to discomfort, pruritus, skin irritation and edema.^70^^,^^71^ Adherence to compressive treatment over a four-week period is as low as 40%,^70^ thus compromising the accuracy of any estimates of treatment effect.^63^ To date, the causal relationship between symptoms and varicose veins remains uncertain.^72^ These factors may lead to many unnecessary treatments. On the positive side, stockings can be used to reduce the incidence of hematomas and thrombophlebitis^61^ and leg ulcers,^57^ thereby reducing the time taken for healing^73^ and the recurrence rate. However, it is logical to claim that the best intervention should aim to treat the primary cause of leg ulcers. It has been found that surgery is just as effective in healing leg ulcers as are compression stockings, and it additionally reduces the recurrence rate.^60^ This should always be considered in cases of leg ulcers that are associated with varicose disease.^74^ Even though use of stockings in the postoperative period has been recommended by some authors,^63^ the effectiveness of this intervention was not found to be superior over the short term (seven days) or medium term (three weeks).^62^

Regarding the implications for practice of our analysis, the important question to be formulated is how much longer should be waited before the paradigms for varicose vein treatment are changed.^75^ This question remains to be answered, considering the current body of literature. According to Chalmers and Glasziou,^76^ gaps in knowledge occur when study questions are not well formulated, studies are not well designed, studies are not published, or there is still a lack of data on a particular topic. Surgery seems to be the most frequent intervention for varicose vein disease in many countries, but new endovascular techniques may provide an alternative for reducing costs and risks. Nonetheless, the studies underpinning these observations have presented serious limitations that have had a negative impact on the strength of the derived evidence, due to the indirectness, low number of events and small sample sizes of these studies.

## CONCLUSIONS

There is evidence of low to moderate quality to suggest that minimally invasive treatments, including foam sclerotherapy, laser and radiofrequency are comparable to conventional surgery, regarding their effectiveness and safety in treating lower-limb varicose veins.
